# “PEOPLE DON'T LIVE IN A VACUUM”: CO-DEVELOPING A BRAIN HEALTH PILOT PROGRAM IN THE COMMUNITY THROUGH CITIZEN SCIENCE

**DOI:** 10.1093/geroni/igac059.1581

**Published:** 2022-12-20

**Authors:** Claire Wang, Daniel Rong Yao Gan, Eireann O'Dea, Brain Health in the Community (BHIC) Steering Committee

**Affiliations:** Johns Hopkins University , Baltimore City, Maryland, United States; Simon Fraser University, Vancouver, British Columbia, Canada; Simon Fraser University, North Vancouver, British Columbia, Canada; Simon Fraser University, Vancouver, British Columbia, Canada

## Abstract

The Psychosocial Model of Everyday Cognitive Resilience identifies social identity as an important determinant of older adults’ wellbeing as they experience cognitive decline in community settings. We engaged community-dwelling older adults to assess the model and co-develop programs that address existing gaps through twelve focus groups. N=55 older adults were recruited from various community organizations. Two 1-hour sessions discussed (1) variables that were important to older adults, namely neighbourhood friendship and social experiences, and (2) how these mediated the effects of self-expression, time outdoors, and communal provisions on mental wellbeing. Many participants highlighted the importance of strong friendship for deeper needs such as grief support, whereas others pointed out the relevance of meaningful activities or volunteering opportunities for a sense of purpose. Overall, a speed-friending program with an emphasis on listening was desirable for connecting and contributing socioemotionally to develop “happy medium” friendships, while piloting evidence-based interventions for brain health.

